# Comparing outcomes of total hip arthroplasty versus hemiarthroplasty in neck of femur fracture patients: an Australian registry study

**DOI:** 10.1007/s00068-023-02305-w

**Published:** 2023-06-24

**Authors:** James Onggo, Mithun Nambiar, Catherine McDougall, Raphael Hau, Sina Babazadeh

**Affiliations:** 1 Department of Orthopaedic Surgery, Box Hill Hospital, 8 Arnold Street, Box Hill, Melbourne, VIC 3128 Australia; 2 Faculty of Medicine, Nursing and Health Sciences, Monash University, Melbourne, Australia; 3 Department of Orthopaedic Surgery, Metro North Hospital and Health Service, Brisbane, Australia; 4 Faculty of Medicine, University of Queensland, Brisbane, Australia

**Keywords:** Neck of femur, Total hip arthroplasty, Hemiarthroplasty, Outcome, Discharge destination

## Abstract

**Purpose:**

To determine discharge outcomes of displaced subcapital NOF patients who were from home, with intact pre-operative cognition, ASA 1 or 2 and independent walkers treated with either THA or hemiarthroplasty.

**Methods:**

A retrospective registry study was performed using data from the Australia and New Zealand Hip Fracture Registry (ANZHFR). Institutional ethics approval was obtained prior to commencement. Hip fracture registry records between 1st January 2016 and 31st January 2020 were reviewed.

**Results:**

A total of 930 patients with complete records were identified and included. There were 602 THA and 328 hemiarthroplasty patients. Using multivariate analysis, pre-operative factors associated with THA include younger age (OR = 0.90 for every year older, *p* < 0.001), females (*p* = 0.043), private admissions (OR = 1.62, *p* = 0.028) and receiving pre-operative geriatric assessment (OR = 1.89, *p* = 0.002). Delay to theatre due to not being fit for surgery was associated with not receiving THA (OR = 0.21, *p* < 0.001). THA resulted in a shorter total hospital length of stay (MD = 7.24, *p* < 0.001), higher likelihood of being discharged home (OR = 1.88, *p* < 0.001) and lower likelihood of being discharged to a residential aged care facility (OR = 0.32, *p* = 0.019).

**Conclusion:**

Displaced subcapital NOF patients who were admitted from home, had intact pre-operative cognition, ASA 1 or 2, independent walkers and had THAs, had shorter total hospital length of stay, were more likely to be discharged home directly and less likely to end up in residential aged care facilities compared to those undergoing hemiarthroplasty.

## Introduction

Displaced subcapital neck of femur fractures (NOF) are associated with high morbidity and mortality rates, along with a reduction in quality of life [1]. These injuries are often treated with total hip arthroplasty (THA) or hemiarthroplasty as blood supply to the femoral head is often compromised. The aims of arthroplasty surgery are to reduce pain and allow patients to weight bear and mobilise as soon as possible after surgery. This potentially reduces the risks of mortality and morbidities associated with non-operative management including pressure sores, infections and functional decline [1, 2].

There have been ongoing debates as to which patients should receive THA over hemiarthroplasty, with the former often offered to patients with higher premorbid functional level [3]. Considerations include medical comorbidities, functional level, cognition status and physical demands of the daily activities of living of the patient [4]. There are still concerns about the use of THA over hemiarthroplasty including greater associated surgical morbidity and higher complication rates including dislocations which may lead to unplanned procedures to reduce or revise the prosthesis [1, 5, 6].

A previous meta-analysis reported THA to be superior to hemiarthroplasty and recommended for patients with a life expectancy > 4 years and in patients younger than 80 years to receive THA [5]. However, other studies also reported similar complication rates up to 5 years and a small clinically insignificant benefit in health-related quality of life with THA [6, 7]. Following a study examining the trends in NOF treatment with arthroplasty from 2004 to 2013, there has been a significant increase in the use of THA, with patients receiving THA being less likely to sustain the same admission mortality and more likely to discharge home when compared to hemiarthroplasty patients [8].

The purpose of this study is to investigate the discharge outcomes of THA versus hemiarthroplasty in displaced subcapital NOF patients who had American Society of Anaesthesiologists (ASA) scores 1 or 2, intact pre-operative cognition status, from home and were independent ambulators from an Australian registry perspective. Our hypothesis is that this subgroup of patients receiving THA would have a shorter length of stay and be more likely to be discharged home directly.

## Methods

The Australia and New Zealand Hip Fracture Registry (ANZHFR) is a clinical quality registry that began data collection in 2016 for patients admitted to hospital with a broken hip in Australia and New Zealand. The ANZHFR is designed to allow hospitals to audit the care they provide against key markers of safe and high-quality care. The data can then be used to improve clinical performance, analyse national trends, advocate for clinical care improvement, and optimise patient outcomes. The registry is managed by a group of clinicians and experts in the field with representation from a number of key professional organisations. More than 93 hospitals across Australia and New Zealand contributed data to the ANZHFR in 2021, and a total of more than 83,000 hip fracture presentations have been collected since its inception.

A retrospective registry study was performed using data from the ANZHFR. Institutional ethics approval was obtained prior to commencement. Hip fracture registry records between 1st January 2016 and 31st January 2020 were reviewed. Only patients with displaced subcapital NOF managed with either a THA or a hemiarthroplasty, who had ASA scores 1 or 2, intact pre-operative cognition status, from home, independent ambulators and had complete records were selected. These selection criteria were used to decrease the heterogenicity of the data and selection bias for treatment, focussing on the high-functioning cohort of patients.

Basic demographics and pre-operative factors including age, sex, admission type, pre-operative medical assessment, side of fracture, delay to surgery, reasons for the delay, type of anaesthesia, type of analgesia nerve block, type of stem fixation, post-operative weightbearing instructions, first-day mobilisation and geriatric medicine assessment. Primary outcome measures include acute ward discharge destination, hospital discharge destination, and acute and hospital length of stay. Acute length of stay refers to the duration in which the patient spends in the acute surgical ward. Hospital length of stay refers to the acute length of stay plus the time spent in inpatient rehabilitation and respite programmes.

### Statistical analysis

Independent samples *t* tests and Chi-square tests were used to compare continuous and categorical variables, as appropriate. Binary and multiple logistic regression models were used to identify pre-operative and post-operative factors associated with THA patients. Multiple logistic regression models allow for the adjustment of potential confounders and allow for numerical representation of the odds ratio associated with THA. Age, sex, type of admission, delays to surgery, pre-operative medical assessment, side of the fracture, type of anaesthesia and analgesia nerve blocks, weightbearing status, first-day mobilisation and assessment by geriatric medicine were included in the multiple logistic regression analysis. All analyses were conducted by using STATA v17 (StataCorp, College Station, TX, USA) [9] with a significance level of *p* < 0.05.

## Results

A total of 930 patients met the inclusion criteria, with 602 receiving THA and 328 receiving hemiarthroplasties. Using univariate analysis, pre-operative factors associated with having a THA included the patient being younger (MD = 7.2 years, *p* < 0.001), and non-public admission (48.5% vs 59.8%, *p* = 0.001). THA patients also had fewer delays to surgery (13.1% vs 20.7%, *p* = 0.002). Post-operatively, THA patients were more likely to be directly discharged home (52.3% vs 28.0%, *p* < 0.001) and less likely to be discharged to a residential aged care facility eventually (1.5% vs 5.8%, *p* = 0.001). THA patients had a similar acute length of stay (MD = 1.08 days, *p* = 0.073) but a much shorter total hospital length of stay (MD = 7.24 days, *p* < 0.001) (Table 1).

**Table 1:** Perioperative details of NOF patients receiving hemiarthroplasty and THA

	Hemi (*n* = 328)	THA (*n* = 602)	*p* value
Age	79.4 ± 8.7	72.2 ± 8.3	** < 0.001**
Sex			0.1
Male	89 (27.1)	134 (22.3)	
Female	238 (72.6)	468 (77.7)	
Others	1 (0.3)	0 (0.0)	
Admission type			**0.001**
Public	196 (59.8)	292 (48.5)	
Private	57 (17.4)	108 (17.9)	
Overseas	9 (2.7)	11 (1.8)	
Not known	66 (20.1)	191 (31.7)	
Pre-operative medical assessment			0.406
Nil	98 (29.9)	206 (34.2)	
Geriatrician	166 (50.6)	298 (49.5)	
Physician	60 (18.3)	89 (14.8)	
GP	0 (0.0)	2 (0.3)	
Specialist nurse	1 (0.3)	4 (0.6)	
Not known	3 (0.9)	3 (0.5)	
Side of fracture			0.69
Left	181 (55.2)	324 (53.8)	
Right	147 (44.8)	278 (46.2)	
Delay to surgery			**0.002**
No delay, < 48 h	260 (79.3)	523 (86.9)	
Unknown	4 (1.2)	5 (0.8)	
Reasons for delay			
Medical reason	9 (2.7)	4 (0.7)	0.0825
Anticoagulation	7 (21.3)	6 (1.0)	0.57
Theatre availability	36 (11.0)	42 (7.0)	0.952
Surgeon availability	1 (0.3)	6 (1.0)	0.081
Delayed diagnosis	2 (0.6)	1 (0.2)	0.476
Others	9 (2.7)	15 (2.5)	0.337
Type of anaesthesia			0.772
General	170 (51.8)	306 (50.8)	
Spinal/regional	117 (35.7)	221 (36.7)	
General and spinal/regional	40 (12.2)	69 (11.5)	
Other	0 (0.0)	2 (0.3)	
Not known	1 (0.3)	4 (0.7)	
Analgesia nerve block			0.8
Nerve block before OT	149 (45.4)	249 (41.4)	
Nerve block in OT	42 (12.8)	85 (14.1)	
Both	84 (25.6)	164 (27.2)	
Neither	39 (11.9)	73 (36.5)	
Unknown	14 (4.3)	31 (5.1)	
Type of stem fixation			0.152
Cemented stem	288 (87.8)	514 (85.4)	
Uncemented stem	40 (12.2)	88 (14.6)	
Postoperative weight bearing instructions			0.088
Unrestricted weight bearing	324 (98.8)	580 (96.3)	
Restricted/non weight bearing	4 (1.2)	20 (3.3)	
Not known	0 (0.0)	2 (0.3)	
First day mobilisation			0.466
Opportunity given	312 (95.1)	571 (94.9)	
Opportunity not given	14 (4.3)	30 (5.0)	
Unknown	2 (0.6)	1 (0.2)	
Assessed by geriatric medicine			**0.001**
Yes	23 (7.0)	95 (15.8)	
No	303 (92.4)	501 (83.2)	
No service available	2 (0.6)	3 (0.5)	
Unknown	0 (0.0)	3 (0.5)	
Acute ward discharge destination			** < 0.001**
Private residence	92 (28.0)	315 (52.3)	
Residential aged care facility	2 (0.6)	2 (0.3)	
Rehabilitation unit public	169 (51.5)	191 (31.7)	
Rehabilitation unit private	47 (14.3)	65 (10.8)	
Other hospital/ward/specialty	18 (5.5)	26 (4.3)	
Deceased	0 (0.0)	0 (0.0)	
Short term care in residential care facility	0 (0.0)	1 (0.2)	
Other	0 (0.0)	2 (0.3)	
Hospital discharge destination			**0.001**
Private residence	277 (84.5)	529 (87.9)	
Residential age care	19 (5.8)	9 (1.5)	
Deceased	2 (0.6)	0 (0.0)	
Other	26 (7.9)	52 (8.6)	
Unknown	4 (1.2)	12 (2.0)	
Acute length of stay	7.54 ± 11.02	6.46 ± 7.24	0.073
Hospital length of stay	19.04 ± 17.86	11.80 ± 12.15	** < 0.001**
Death during acute admission	0 (0.0)	0 (0.0)	**–**

Continuous variables presenting in terms of mean ± standard deviation, while non-continuous variable is presented in terms *n* (percentage)

Bold indicates a statistically significant result

Using multivariate analysis with adjustments to confounders, pre-operative factors predictive of THA can be found in Table 2. It was noted that after multivariate analysis, being female (OR = 1.44, 95% CI 1.01–2.06, *p* = 0.043), having preoperative geriatric assessment (OR = 1.89, 95% CI 1.26–2.83, *p* = 0.002) and less delays to surgery for medical reasons (OR = 0.21, 95% CI 0.05–0.80, *p* = 0.023) were additional factors associated with patients receiving THA. Younger age and private admissions remained as predictive factors for receiving THA after multivariate analysis. Other reasons for delays to surgery, type of anaesthesia and type of nerve block were not associated with patients receiving THA. (Table 2).

**Table 2:** Multivariate analysis to investigate predictive factors of receiving THA for NOF

Predictive factors for THA	Adjusted odds ratio	95% Confidence interval	*p* value
Age	**0.90**	**0.88–0.92**	** < 0.001**
Female vs male	**1.44**	**1.01–2.06**	**0.043**
Private vs public	**1.62**	**1.06**–**2.51**	**0.028**
Overseas visitors vs public	0.42	0.16–1.12	0.084
Preoperative assessment by geriatrician vs nil	**1.89**	**1.26–2.83**	**0.002**
Preoperative assessment by physician vs nil	1.13	0.68–1.87	0.627
Fracture side right vs left	1.11	0.81–1.53	513
Delay due to patient medically unfit vs no delay	**0.21**	**0.05–0.80**	**0.023**
Delay due to issues with anticoagulation vs no delay	0.67	0.20–2.30	0.527
Delay due to theatre availability vs no delay	0.59	0.34–1.02	0.063
Delay due to surgeon availability vs no delay	2.02	0.21–19.97	0.546
Delay due to delayed diagnosis of hip fracture vs no delay	0.63	0.24–1.65	0.346
other type of delay vs no delay	0.33	0.07–1.50	0.152
Spinal vs GA	1.17	0.82–1.66	0.387
GA + Spinal vs GA	0.86	0.52–1.43	0.568
nerve block in OT Vs before OT	1.03	0.63–1.70	0.903
Both vs before OT	1.41	0.95–2.09	0.09
neither vs before OT	0.82	0.49–1.40	0.469

Bold indicates a statistically significant result

Patients receiving THA were associated with more direct discharge home (OR = 1.88, 95% CI 1.33–2.66, *p* < 0.001), less hospital discharge to nursing homes (OR = 0.32, 95% CI 0.12–0.83, *p* = 0.019) and shorter hospital length of stay (MD = 7.24 days, 95% CI 5.30–9.19, *p* < 0.001). There was a similar acute length of stay for both groups (Table 3).

**Table 3:** Multivariate analysis investigating predictive factors of direct discharge home and final discharge to residential aged care facilities

	Direct discharge home	Final discharge NH
Receive THA	**OR = 1.88, 95% CI 1.33–2.66, *p* < 0.001**	**OR = 0.32, 95% CI 0.12–0.83, *p* = 0.019**
Age	**OR = 0.91, 95% CI 0.89–0.93, *p* < 0.001**	OR = 1.05, 95% CI 1.00–1.10, *p* = 0.071
Female vs male	**OR = 0.64, 95% CI 0.45–0.91, *p* = 0.012**	OR = 0.86, 95% CI 0.34–2.17, *p* = 0.754
Private vs public	OR = 0.70, 95% CI 0.46–1.07, *p* = 0.098	OR = 2.36, 95% CI 0.82–6.79, *p* = 0.111
Overseas visitor vs public	**OR = 3.67, 95% CI 1.11–12.18, *p* = 0.001**	–
Preoperative assessment by geriatrician vs nil	OR = 0.74, 95% CI 0.52–1.07, *p* = 0.112	OR = 1.64, 95% CI 0.61–4.38, *p* = 0.325
Preoperative assessment by physician vs nil	OR = 1.14, 95% CI 0.70–1.84, *p* = 0.604	OR = 0.60, 95% CI 0.13–2.74, *p* = 0.141
Fracture side right vs left	OR = 0.90, 95% CI 0.66–1.21, *p* = 0.489	OR = 1.74, 95% CI 0.77–3.91, *p* = 0.181
Delay due to patient medically unfit vs no delay	OR = 1.27, 95% CI 0.37–4.36, *p* = 0.701	**OR = 6.43, 95% CI 1.43–28.96, *p* = 0.015**
Delay due to issues with anticoagulation vs no delay	OR = 1.01, 95% CI 0.24–4.33, *p* = 0.988	–
Delay due to theatre availability vs no delay	OR = 0.77, 95% CI 0.44–1.33, *p* = 0.342	–
Delay due to surgeon availability vs no delay	OR = 1.50, 95% CI 0.31–7.13, *p* = 0.614	–
Delay due to delayed diagnosis of hip fracture vs no delay	OR = 7.09, 95% CI 0.40–124.13, *p* = 0180	–
Other type of delay vs no delay	OR = 0.87, 95% CI 0.35–2.15, *p* = 0.756	OR = 1.32, 95% CI 0.14–12.51, *p* = 0.803
Spinal vs GA	OR = 0.96, 95% CI 0.69–1.32, *p* = 0.788	OR = 0.33, 95% CI 0.12–0.89, *p* = 0.028
GA + spinal vs GA	OR = 0.63, 95% CI 0.38–1.03, *p* = 0.064	–
Nerve block in OT Vs before OT	OR = 1.26, 95% CI 0.80–1.99, *p* = 0.327	–
Both vs before OT	OR = 0.76, 95% CI 0.53–1.11, *p* = 0.158	–
Neither vs before OT	OR = 0.81, 95% CI 0.49–1.31, *p* = 0.383	–
Restriction weightbearing vs nil restriction	OR = 0.60, 95% CI 0.22–1.60, *p* = 0.301	–
Not given 1st day mobilisation	OR = 1.35, 95% CI 0.64–2.84, *p* = 0.434	OR = 3.16, 95% CI 0.69–14.34, *p* = 0.137

Bold indicates a statistically significant result

A younger age (OR = 0.91 for every year older, 95% CI 0.89–0.93, *p* < 0.001), males (OR = 0.91 for females, 95% CI 0.45–0.91, *p* = 0.012) and overseas visitors (OR = 3.67, 95% CI 1.11–12.18, *p* = 0.001) were also positively associated with patients having direct discharge home, while delay to surgery due to the patient being medically unfit (OR = 6.43, 95% CI 1.43–28.96, *p* = 0.015) was positively associated with patients having final discharge to residential aged care facilities.

## Discussion

Displaced subcapital NOF patients with ASA scores 1 or 2, from home, who were independent ambulators and treated with THA were associated with a shorter hospital length of stay, higher likelihood of direct discharge home and less likelihood of discharge to a nursing home. Our results suggest that receiving THA for this high-functioning NOF patient subgroup may bring potential benefits of maintaining independence and consequent reduction in strain to the health budget.

While THA is able to better restore the anatomical and biomechanical features of the hip joint and femoral neck than hemiarthroplasties [10], one of the arguments against it in this patient subgroup is the risk of dislocations resulting in the need for secondary procedures to reduce or revise the prosthesis [11]. However, this was disputed by a multicentre randomized controlled trial that reports no significant difference in the incidence of secondary procedures between THA and hemiarthroplasties [7]. Another recent retrospective study also advocated for THA as a treatment of choice, especially for healthy and active patients with no increase in mortality, morbidity, bleeding or dislocation rate when compared to bipolar hemiarthroplasties [12].

Although trends in the United States did reveal similar results to the current study, their results did not specify this subgroup of high-functioning patients and hence would be confounded by the more unwell patients receiving hemiarthroplasties and requiring more medical support peri-operatively. This could have severely skewed their results [8]. Very often, determining what surgery patients receive is very subjective and often depends on age, comorbidities and mobility, leading to selection bias. To overcome this, we selected a rather homogeneous healthy subgroup of patients who would similarly be potential candidates for THA in the setting of osteoarthritis. This builds a stronger basis of consideration to offer these NOF patients with a THA, especially when significant benefits in terms of discharge disposition and hospital length of stay are reported.

With an ever-increasing demand for healthcare services due to an aging population and the current healthcare climate, early and direct safe discharge home is a crucial mean for efficient use of resources. A previous retrospective cohort study by Salar et al. [13] reported independent variables associated with a higher likelihood of discharge home to include patients walking independently outdoors, no use of walking aids, intact cognitive status, absence of some comorbidities, and no assistance required with basic activities of daily living and intracapsular fractures. Salar et al. [13] reported a 52% direct home discharge rate in a cohort of 6742 patients, which further corroborates our results of 52.3% direct home discharge. Furthermore, another registry study reported that regardless of dementia, delirium, hypotension, preinjury ambulation or residence, early compared to late mobilisation increased the likelihood of hospital discharge by 30 days postoperatively in NOF patients [14]. Both studies strongly suggest that our patient subgroup are good candidates for home discharge should they receive early mobilisation. While both arms have approximately 95% of patients receiving early mobilisation, there is a still large discrepancy in the home discharge rate of THA versus hemiarthroplasty. Perhaps, the type of surgery performed plays an instrumental role, though this was not shown in the results reported by Salar et al. [13]

A recent cost-effectiveness study found better quality-adjusted life years in patients with earlier discharge [15]. Early discharge home after THA may also translate to potential benefits for the patient and health system and is a key surgical goal. A previous meta-analysis comparing home discharge and inpatient rehabilitation for elective THA found that inpatient rehabilitation was associated with higher risks of complications and readmission compared to home discharge [16]. Moreover, up to 55% of the costs of elective and emergency THA stem from post-acute care, with large variability dependent on patient’s discharge destination [17]. Hence, early home discharge with home intervention is a viable target for cost savings, potentially reducing up to 28% unit cost reduction in THA for our increasingly exorbitant health system [15, 17].

We noted that patients whose surgeries were delayed due to being medically unfit were associated with less likelihood to receive THA. Hemiarthroplasty may be preferentially chosen in these patients as THA is thought to increase risk due to longer operative time, blood loss, postoperative transfusion, medical complications, and dislocation risks [11]. However, recent comparisons between THA and hemiarthroplasties have shown no differences in terms of functional outcomes and complications [6, 7, 12]. This could be the result of increasing worldwide emphasis on clinical care standards for managing hip fracture patients, with a standardised multidisciplinary approach, preoperative medical optimisation, perioperative multimodal analgesia and nerve blocks [18, 19]. Furthermore, with advances in surgical techniques and improvements in prosthesis options such as large femoral heads and dual mobility constructs, surgical risks with THA are significantly reduced [11, 20].

Unfortunately, these patients with delayed surgery for medical reasons were also associated with a higher likelihood of discharge to a residential aged care facility. This suggests that some of the medical comorbidities in our patient cohort could have persistent debilitating effects that affect overall long-term physical and rehabilitation capacity. It is obvious that there are still limitations on how much preoperative optimisation could be offered to restore normal physiology. Hence, clinical judgement on the prognosis of the patient along with informed decision making with the patient and family members remain crucial considerations for the type of surgery performed.

There are some limitations to this study. Being a retrospective registry study, not all biases can be completely excluded. Furthermore, despite having over 40,000 records available, only 930 complete records in this subgroup were included, suggesting the possibility of a heterogenous data collection methodology. While an attempt to reduce selection bias has been performed, there is only a fair interrater reliability (Kappa = 0.40) of the ASA score among Australian anaesthetists [21]. Hence, perhaps a more comprehensive and reliable evaluation of the physical status and health of patients may be required for future entries. The ANZHFR data also did not collect other pertinent clinical information such as conversion to THA or dislocation rates that could also help supplement clinical decision making when considering between THA and hemiarthroplasties.

## Conclusion

Healthy and high functioning NOF patients treated with THA were more likely to be discharged home directly, were less likely to end up in residential aged care facilities and had shorter total hospital length of stay compared to patients treated with hemiarthroplasties.

## References

[1] Bhandari M, Swiontkowski M (2017). Management of acute hip fracture. N Engl J Med.

[2] Kim SJ, Park HS, Lee DW (2020). Outcome of nonoperative treatment for hip fractures in elderly patients: a systematic review of recent literature. J Orthop Surg (Hong Kong).

[3] National Institute for Health and Care Excellence (2023) The management of hip fracture in adults [NICE Guideline CG124]. Available from: https://www.nice.org.uk/guidance/cg124/evidence/full-guideline-pdf-183081997. 31909929

[4] Studnicka KJ, Kumar G (2021). Total hip replacement for displaced intracapsular neck of femur fracture. Are current guidelines appropriate for all patients? Five-year retrospective analysis of 315 cases. Injury.

[5] Lewis DP, Wæver D, Thorninger R, Donnelly WJ (2019). Hemiarthroplasty vs total hip arthroplasty for the management of displaced neck of femur fractures: a systematic review and meta-analysis. J Arthroplasty.

[6] Ekhtiari S, Gormley J, Axelrod DE, Devji T, Bhandari M, Guyatt GH (2020). Total hip arthroplasty versus hemiarthroplasty for displaced femoral neck fracture: a systematic review and meta-analysis of randomized controlled trials. J Bone Jt Surg Am.

[7] Bhandari M, Einhorn TA, Guyatt G, Schemitsch EH, Zura RD, Sprague S (2019). Total hip arthroplasty or hemiarthroplasty for hip fracture. N Engl J Med.

[8] Stronach BM, Bergin PF, Perez JL, Watson S, Jones LC, McGwin G (2020). The rising use of total hip arthroplasty for femoral neck fractures in the United States. Hip Int.

[9] StatCorp (2021). Stata statistical software: release 17.

[10] Ossendorf C, Scheyerer MJ, Wanner GA, Simmen HP, Werner CM (2010). Treatment of femoral neck fractures in elderly patients over 60 years of age—which is the ideal modality of primary joint replacement?. Patient Saf Surg.

[11] Goru P, Haque S, Verma GG, Mustafa A, Hamed M, Ismail M (2022). Dislocation of total hip replacement in femoral neck fracture: do surgical approach and dual mobility implant matter?. Cureus..

[12] Rotini M, Farinelli L, Natalini L, De Rosa F, Politano R, Cianforlini M (2022). Is dual mobility total hip arthroplasty surgery more aggressive than hemiarthroplasty when treating femoral neck fracture in the elderly? A multicentric retrospective study on 302 hips. Geriatr Orthop Surg Rehabil.

[13] Salar O, Baker PN, Forward DP, Ollivere BJ, Weerasuriya N, Moppett IK (2017). Predictors of direct home discharge following fractured neck of femur. Ann R Coll Surg Engl.

[14] Sheehan KJ, Goubar A, Martin FC, Potter C, Jones GD, Sackley C (2021). Discharge after hip fracture surgery in relation to mobilisation timing by patient characteristics: linked secondary analysis of the UK National Hip Fracture Database. BMC Geriatr.

[15] Shi Y, Zhu P, Jia J, Shao Z, Yang S, Chen W (2022). Cost-effectiveness of same-day discharge surgery for primary total hip arthroplasty: a pragmatic randomized controlled study. Front Public Health.

[16] Onggo JR, Onggo JD, De Steiger R, Hau R (2019). The efficacy and safety of inpatient rehabilitation compared with home discharge after hip or knee arthroplasty: a meta-analysis and systematic review. J Arthroplasty.

[17] Sigurdsson E, Siggeirsdottir K, Jonsson H, Gudnason V, Matthiasson T, Jonsson BY (2008). Early discharge and home intervention reduces unit costs after total hip replacement: results of a cost analysis in a randomized study. Int J Health Care Finance Econ.

[18] British Orthopaedic Association BGS (2007) The blue book: the care of patients with fragility fracture.

[19] Care ACoSaQiH (2016). Hip fracture clincial care standard.

[20] Ertaş ES, Tokgözoğlu AM (2021). Dislocation after total hip arthroplasty: does head size really matter?. Hip Int.

[21] Riley R, Holman C, Fletcher D (2014). Inter-rater reliability of the ASA physical status classification in a sample of anaesthetists in Western Australia. Anaesth Intensive Care.

